# Causal inference multiple imputation investigation of the impact of cannabinoids and other substances on ethnic differentials in US testicular cancer incidence

**DOI:** 10.1186/s40360-021-00505-x

**Published:** 2021-07-11

**Authors:** Albert Stuart Reece, Gary Kenneth Hulse

**Affiliations:** 1 Division of Psychiatry, University of Western Australia, Crawley, Western Australia 6009 Australia; 2 School of Medical and Health Sciences, Edith Cowan University, Joondalup, Western Australia 6027 Australia

**Keywords:** Testicular cancer, Cannabis, Other drugs, Ethnicity, Pathways, Mechanisms, Casual inference

## Abstract

**Background:**

Ethnic differences in testicular cancer rates (TCRs) are recognized internationally. Cannabis is a known risk factor for testicular cancer (TC) in multiple studies with dose-response effects demonstrated, however the interaction between ancestral and environmental mutagenic effects has not been characterized. We examined the effects of this presumed gene-environment interaction across US states.

**Methods:**

State based TCR was downloaded from the Surveillance Epidemiology and End Results (SEER) website via SEERStat. Drug use data for cigarettes, alcohol use disorder, analgesics, cannabis and cocaine was taken from the National Survey of Drug Use and Health a nationally representative study conducted annually by the Substance Abuse and Mental Health Services Administration (SAMHSA) with a 74.1% response rate. Cannabinoid concentrations derived from Drug Enforcement Agency publications. Median household income and ethnicity data (Caucasian-American, African-American, Hispanic-American, Asian-American, American-Indian-Alaska-Native-American, Native-Hawaiian-Pacific-Islander-American) was from the US Census Bureau. Data were processed in R using instrumental regression, causal inference and multiple imputation.

**Results:**

1975–2017 TCR rose 41% in African-Americans and 78.1% in Caucasian-Americans; 2003–2017 TCR rose 36.1% in Hispanic-Americans and 102.9% in Asian-Pacific-Islander-Americans. Ethnicity-based scatterplot-time and boxplots for cannabis use and TCR closely mirrored each other. At inverse probability-weighted interactive robust regression including drugs, income and ethnicity, ethnic THC exposure was the most significant factor and was independently significant (β-estimate = 4.72 (2.04, 7.41), *P* = 0.0018). In a similar model THC, and cannabigerol were also significant (both β-estimate = 13.87 (6.33, 21.41), *P* = 0.0017). In additive instrumental models the interaction of ethnic THC exposure with Asian-American, Hispanic-American, and Native-Hawaiian-Pacific-Islander-American ethnicities was significant (β-estimate = − 0.63 (− 0.74, − 0.52), *P* = 3.6 × 10^− 29^, β-estimate = − 0.25 (− 0.32, − 0.18), *P* = 4.2 × 10^− 13^, β-estimate = − 0.19 (− 0.25, − 0.13), *P* = 3.4 × 10^− 9^). After multiple imputation, ethnic THC exposure became more significant (β-estimate = 0.68 (0.62, 0.74), *P* = 1.80 × 10^− 92^). 25/33 e-Values > 1.25 ranging up to 1.07 × 10^5^. Liberalization of cannabis laws was linked with higher TCR’s in Caucasian-Americans (β-estimate = 0.09 (0.06, 0.12), *P* = 6.5 × 10^− 10^) and African-Americans (β-estimate = 0.22 (0.12, 0.32), *P* = 4.4 × 10^− 5^) and when dichotomized to illegal v. others (t = 6.195, *P* = 1.18 × 10^− 9^ and t = 4.50, *P* = 3.33 × 10^− 5^).

**Conclusion:**

Cannabis is shown to be a TC risk factor for all ethnicities including Caucasian-American and African-American ancestries, albeit at different rates. For both ancestries cannabis legalization elevated TCR. Dose-response and causal relationships are demonstrated.

## Background

Testicular Cancer (TC) is the commonest cancer in men aged 15–44 years, and rates have increased two to three times in many nations in recent decades [1]. Indeed TC is the leading cause of individual ‘years of life lost’ of any adult cancer [1].

Both genetic and environmental factors are believed to be significant, with 25% of the risk ascribed to genetic factors [1, 2]. This includes eight to ten- fold elevation in risk of brothers of cases, and four- to six- fold elevation in their sons [1]. 2–5% of cases are bilateral [1, 2]. Well-described differing rates by ethnicity have also been reported from many continents including Europe, New Zealand and USA [3–5], with a twenty-fold variation in TC rates (TCRs) in nationals of northern Europe (Denmark and Norway) compared to various African nations [1, 5, 6].

Data from the US SEER*Explorer website reveals that the all-age all-stages age-adjusted TCR in American males rose 83.45% from 3.4415 to 6.3136 / 100,000 in the years 1976–2017 [3]. The commonest age for TC is in males aged 30–34 years which is represented in the official statistics by the 15–39 year age group whose age-adjusted rate rose 92.16% from 6.29 to 12.09 /100,000 from 1975 to 2017 across all ethnicities combined [3].

However, these US TCR trends for “all ethnicities combined” conceal vastly different rates of TCR amongst different ethnic groups. For example amongst the US 15–39 year age-group TCR for Caucasian-Americans vs. African-Americans for 1975 was 7.01, and 3.09/100,000 respectively. However, this TCR for each ethnic group has not remained static over time.

Amongst the 15–39 year age-group the TCR amongst Caucasian-Americans rose 111.61% from 7.01 to 14.81 / 100,000 across the years 1975–2016. In contrast, amongst African-Americans in this age group the TCR *fell* 14.24% from 3.09 to 2.65/100,000. Accordingly across this time-period the mean TCR in Caucasian-Americans increased to a mean of 11.7 (+ 0.3374 S.E.M.), whereas in African Americans decreased to 2.82 (+ 0. 0.0737 S.E.M.) / 100,000. That is the rate of TCR amongst African Americans was decreased to 24.05% of the rate in Caucasian-Americans suggesting a strong gene-environment interaction. SEER*Explorer data show that the rate amongst Caucasian-Americans 15–39 years rose 3.7% annually 1975–1987 and 1.0% from 1987 to 2017 [3]. No annual percent change was listed on this site for African-American ancestry.

Interestingly the nature of the different genetic risk between African-Americans and Caucasian-Americans has been precisely defined as being due to a differing allelic frequency at an anomalous P53 response element on Chromosome 9 which drives cellular proliferation rather than the usual suppression of growth-related activities in cells experiencing genotoxic stress which is more generally associated with P53 activation [7, 8]. This effect is described further in the Discussion.

Curiously, international reports include documentation of very different case rates within the same ethnic group further indicating a strong role for environmental components in TC, with current data suggesting the prospect of one or more gene-environment interactions [7, 9]. For example some ethicities in neighbouring nations across Europe show significantly different rates of TC [1, 5, 6]. Similarly, geographic TC clusters amongst some ethnic populations have also been reported in northern Netherlands [10], while TCR in the Hispanic-American community has been reported as rising most rapidly in recent years [1]. Moreover, a man of Caucasian descent who moves from a low to a high incidence area assumes the risk of the high incidence area in the following generation [1]. This is consistent with data suggesting TC risk is 25% genetic [1, 2], and 75% environmental [7, 9].

It is generally agreed that TC likely results from in utero germ cell anomalies which then become activated by the hormonal surge of adolescence. Important classification and treatment insights have flowed from the increasing recent elucidation of the biology of testicular cancer and its close relationship to disordered primordial germ cell and spermatogonial development often from antenatal germ cell neoplasia in situ (GCNIS) [1, 11–14]. For example, on occasion immune checkpoint inhibition with PD-L1 inhibitors is recommended in selected patients [14].

That higher potency varieties of cannabis have been increasingly used in many countries over the past decades, couple with published data that cannabis exposure is both genotoxic [15], and results in genomic and chromosomal damage [13, 16] raises the possibility that cannabinoid exposure in utero may be a risk factor for TC.

However, notwithstanding the role of genetics and likely in-utero cannabis exposure, all four studies to examine the association of TC and cannabis have noted that personal cannabis use is linked with rising testicular cancer rates [17–20]. Importantly, dose-response effects have been described [17–19] which was also confirmed in meta-analysis [6]. Two meta-analyses have been published on this data which found pooled odds ratios for exposure of 2.59 (95%C.I. 1.60, 4.19) for chronic, current and frequent cannabis use [6] and 1.71 (1.12, 2.60) [21].

Thus while TC may have an genetic component and is believed to arise a result of in utero germ cell anomalies in which cannabis exposure may be a factor, all four studies linking testicular cancer with cannabis use describe personal use in the preceding twenty years. This suggests both that exposure through personal use may be an important environmental component and that the usual pathophysiological processes of testicular cancerogenesis are greatly accelerated by personal cannabinoid exposure. Multiple pathways and mechanisms for cannabis damage to the primordial germ cell are reviewed in the Discussion section.

This evident temporal compression of the natural history of testicular carcinogenesis by cannabis exposure may offer further pathogenetic insights into the accelerated development of this tumour type.

As cannabis use has been linked with TCR in all four studies to examine the association [17–20], and as several reviews of TC epidemiology have noted its likely significance [1, 9, 13, 14, 22], it seemed important to examine the association between cannabis and testicular cancer across both space and time, along with ethnicity and other drug and cannabis exposures. It was also important to determine if any potential relationship satisfied the quantitative criteria for causality in all ethnicities. As data on known risk factors such as cryptorchidism, inguinal herniae, industrial pollution and sedentary lifestyles across space and time was not available to the present investigators it was not possible to include these covariates in the present analysis. The USA formed a suitable setting for this epidemiological investigation as the required data on TCR and other drug and cannabis use by State and different ethnicities over time is readily publicly available.

## Methods

### Data

National data on age-adjusted TCR, age-specific TCR and ethnic TCR were downloaded from the SEER*Explorer website [3]. State based data both as overall age-adjusted TCR and by ethnicity was downloaded using the SEERStat software from the National Program of Cancer Registries (NPCR) and Surveillance Epidemiology and End Results (SEER) Incidence File from the US Cancer Statistics Public Use Database, Submission 2001–2017 [23]. Drug use data for the years 2003–2017 was taken from the Restricted Use Data Analysis System (RDAS) of the Substance Abuse and Mental Health Data Archive (SAMHDA) of the National Survey of Drug Use and Health (NSDUH) from the Substance Abuse and Mental Health Services Administration (SAMHSA) [24]. The major drugs of interest were last year cocaine use (Cocaine), last year non-medical use of pain relievers (Analgesics), last month cigarette use (Cigarettes) and last year abuse or dependence on Alcohol (Alcohol Use Disorder, AUD). Data was supplemented by median household income and state-based ethnicity data from the US Census bureau focussing on Caucasian-American, African-American, Hispanic-American, Asian-American, American Indian / Alaskan Native (AIAN) -American and Native Hawaiian / Pacific Islander (NHPI) -American ethnicities. This ethnicity data was paired as closely as possible with the ethnicity data from the SEER database which sometimes used a slightly different categorization system. Cannabinoid concentration data was obtained from publications of Drug Enforcement Agency listing the various concentrations found in Federal seizures [25–27]. Data relating to the legal status of cannabis in US states was derived from an internet search [28].

### Derived data

Data on intensity of use of cannabis was taken from a variable called “mrjmdays” denoting the number of days in the past month for which cannabis had been used. The responses to this variable are categorized as 0, 1–2, 3–5, 6–19 and 20–30 days per month. For each ethnicity and for each year of the NSDUH the percentage responding in each category was multiplied by the mean number of days in that category and summed to provide an ethnic cannabis use score for that year. This ethnic cannabis use intensity score was then multiplied by the percent of cannabis used last month in that state and the concentration for that year of THC in Federal seizures to provide an estimate of ethnic-specific THC exposure. State-based cannabinoid concentrations were estimated as the product of the concentration of various cannabinoids in Federal seizures multiplied by the last month cannabis use in each State.

### Statistics

Data was processed using R version 4.0.2 and R-Studio 1.3.1093 in October 2020. Data was read in and reconfigured using dplyr from the tidyverse suite [29]. Maps were drawn in ggplot2, sf and RColorBrewer [30–32]. Graphs drawn in Microsoft Excel, ggplot2 and lattice [30, 33]. Data was log transformed as indicated by the results of the Shapiro-Wilks test. All regression models were manually serially reduced by elimination of the least significant term as is performed in the classical technique of model reduction. Mixed effects was conducted using the nlme package [34]. Two-step instrumental variable regression was conducted using the ivreg function from the AER package [35].

Inverse probability weighting was calculated from the ipw package [36] and was used to control for cannabis exposure across all the groups by other substance exposure. This was applied in mixed effects repeated measures, two-step instrumental variable regression and robust regression. Each of these forms of multivariable regression was used for different purposes. Mixed effects and instrumental variable models all provided model standard deviations from which e-Values could be calculated. Robust generalized linear regression from the survey package was used to perform robustified regression [37]. Mixed effects models with state as the random effect were used to account for the repeated measures nature of the data. Instrumental variable regression was used to test directly for cannabinoid effects underlying the effects of the primary covariates as described in the Results section.

SEER data is suppressed for cell counts less than 15 or very low rates. Hence a high rate of missing data was noted particularly for TCR by ethnicity. The problem was most marked for ethnic minorities. This issue was addressed by multiple imputation by chained equations using the mice package in R [38]. In view of the size of the missing data problem 256 imputations were used each with 20 iterations. The initial seed used was 59. The imputation method was by the classification tree (“cart”) method which provided the best fit for the ethnically grouped data. Linear models were used to investigate this data. The results from models were pooled appropriately using Rubin’s rules. Using these techniques the fraction of missing information obtained in simple linear models regressing TCR against ethnic THC exposure was reduced to 2.6%.

e-Values were calculated using the e-Value algorithms of the EValue package [39].

All t-tests were two sided. *P* < 0.05 was considered significant.

### Data sharing and availability

Data has been deposited in the Mendeley data repository along with the software programming code in R and may be found at URL 10.17632/yxy3dg2wt6.1.

### Ethics

All methods were carried out in accordance with relevant guidelines and regulations. This study was approved by the Human Research Ethics Committee of the University of Western Australia on 7th January 2020 RA/4/20/7724.

## Results

The outline plan for the results section is as follows. We first present the univariate ethnic and testicular cancer rate data upon which the analysis rests. We then examine various bivariate relationships including different ethnic time trends. Various forms of multivariable regression are used to determine the impact of multivariate adjustment on the bivariate relationships described. All multivariable models are inverse probability weighted and E-Values are freely used to allow formal causal inferences to be drawn both qualitative and quantitatively. Multiple imputation is employed to complete ethnic TCR data where missing data is severely problematical. Finally the effects of legal status on the TCR in various ethnicities is considered and investigated by the tools of causal inference. Each of these steps in this procedure has several component steps which are included along the way as is mandatory in a formal presentation of such analyses.

### Univariate data

**Fig. 1:**
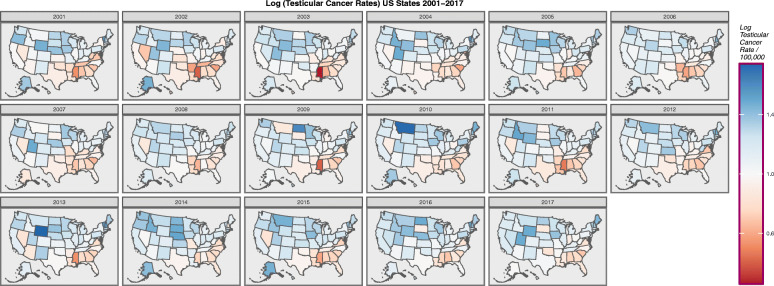
Choropleth map of log(testicular cancer incidence rates) across USA by Year. Maps were drawn in ggplot2, sf and RColorBrewer

Figure 1 shows map-graphically the distribution of the age-adjusted TCRs across USA states over time 2001–2017 across all ages. The maps are notable because cases seem to be most concentrated in the upper midwest and northeast and to be negatively associated with the south-eastern corner.

**Fig. 2:**
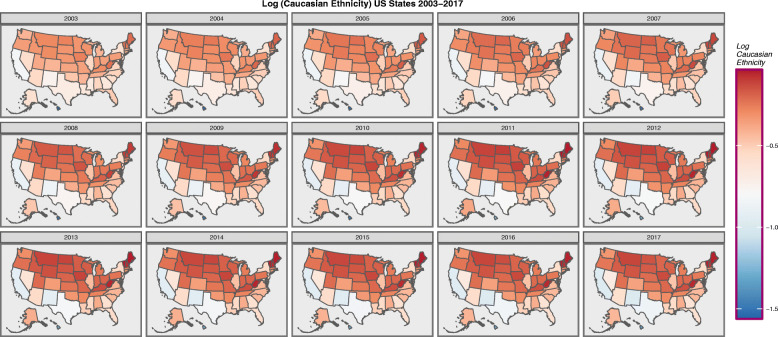
Choropleth map of log(Caucasian-American Population) across USA by Year. Maps were drawn in ggplot2, sf and RColorBrewer

Figure 2 sets out in a similar format the ethnic composition of USA states as the log (Caucasian-American) population.

**Fig. 3:**
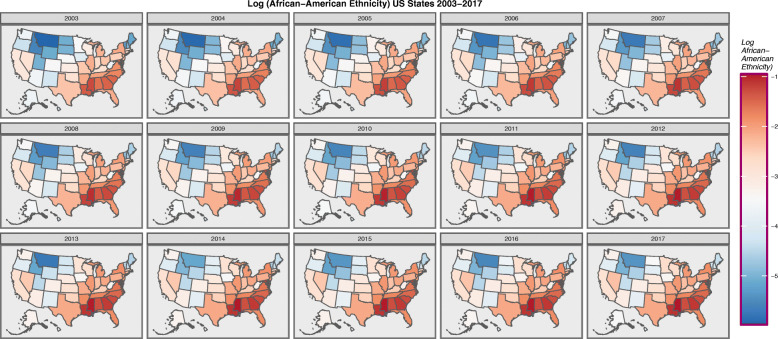
Choropleth map of log(African-American Population) across USA by Year. Maps were drawn in ggplot2, sf and RColorBrewer

Figure 3 does the same for the African-American population.

**Fig. 4:**
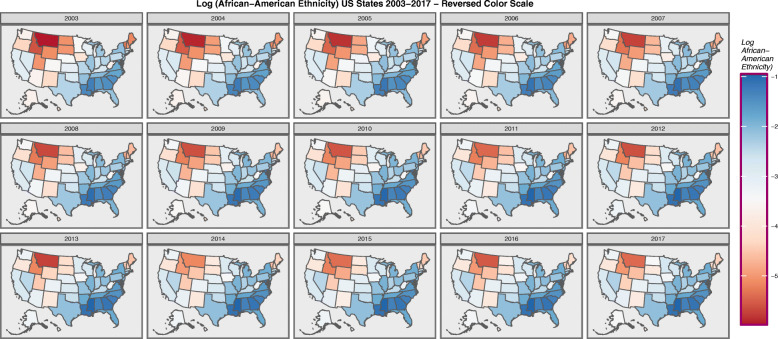
Choropleth map of log(Caucasian-American Population) across USA by Year – Reversed Colouring. Maps were drawn in ggplot2, sf and RColorBrewer

When the colour coding for Fig. 3 is reversed, as shown in Fig. 4, the map resembles Fig. 1 of the TCRs rather closely. This is consistent with the above described preponderance of cases in the Caucasian-American community. One presumes that in these maps ethnicity is acting as a surrogate marker for TC incidence.

**Fig. 5:**
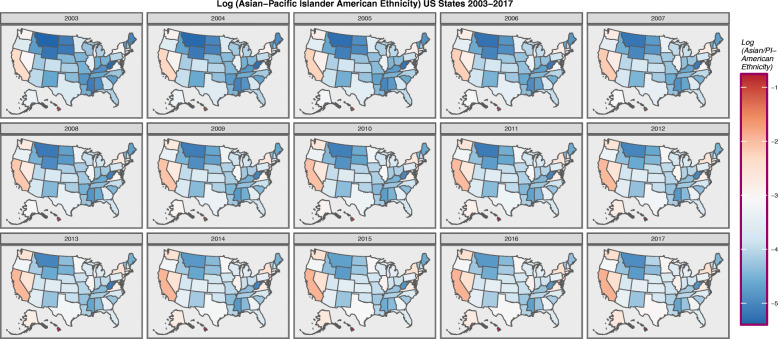
Choropleth map of log(Asian-American Population) across USA by Year. Maps were drawn in ggplot2, sf and RColorBrewer

Figure 5 presents the log of the Asian-American ethnicity.

**Fig. 6:**
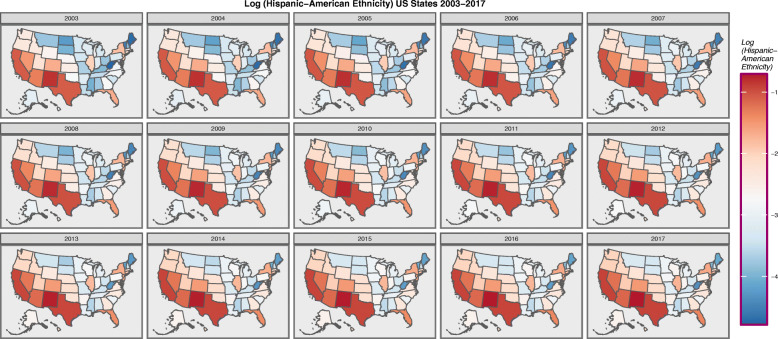
Choropleth map of log(Asian-Pacific Islander-American Population) across USA by Year. Maps were drawn in ggplot2, sf and RColorBrewer

Figure 6 presents the log of the Asian-Pacific Islander -American ethnicity. Hawaii is noted to be a particular hot spot.

**Fig. 7:**
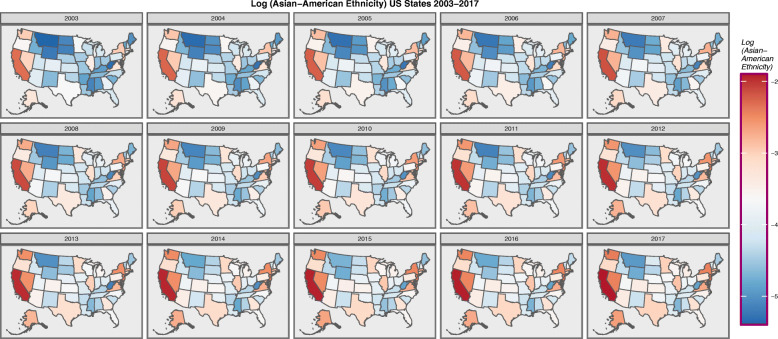
Choropleth map of log(Hispanic-American Population) across USA by Year. Maps were drawn in ggplot2, sf and RColorBrewer

Figure 7 shows the log of the Hispanic-American population density. It seems to be concentrated along the southern border states.

Bivariate Relationships and Ethnic Differentials.

**Fig. 8:**
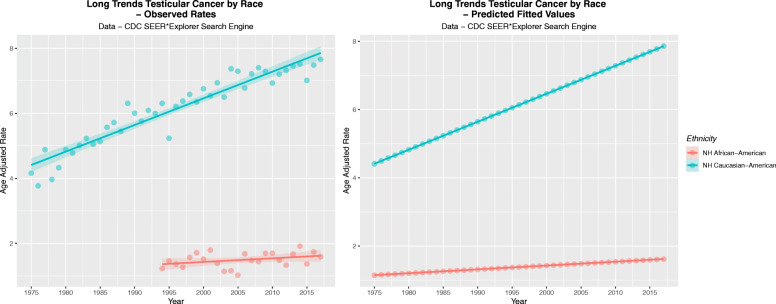
(**A**) Observed and (**B**) Predicted Incidence rates of testicular cancer for two ethnicities

Figure 8A shows the observed age-adjusted TCRs 15–60 years for the Caucasian-American and African-American populations. Figure 8B shows the linear projections of this data.

Table 1 sets out the observed and predicted values from which these graphs were drawn.

**Table 1:** Long Term Testicular Cancer Rates by Ethnicity

Year	Caucasian-American - Observed	African-American - Observed	NH Caucasian-American Predicted	NH African-American Predicted
1975	4.1621		4.4103	1.1457
1976	3.7714		4.4924	1.1570
1977	4.879		4.5744	1.1682
1978	3.9667		4.6565	1.1795
1979	4.3295		4.7385	1.1908
1980	4.8836		4.8205	1.2020
1981	4.7748		4.9026	1.2133
1982	5.0104		4.9846	1.2245
1983	5.2268		5.0667	1.2358
1984	5.0458		5.1487	1.2470
1985	5.1316		5.2307	1.2583
1986	5.567		5.3128	1.2696
1987	5.7151		5.3948	1.2808
1988	5.444		5.4769	1.2921
1989	6.306		5.5589	1.3033
1990	6.0005		5.6410	1.3146
1991	5.7543		5.7230	1.3258
1992	6.0864		5.8050	1.3371
1993	5.9933		5.8871	1.3484
1994	6.305	1.2295	5.9691	1.3596
1995	5.2299	1.4628	6.0512	1.3709
1996	6.2072	1.3649	6.1332	1.3821
1997	6.3764	1.267	6.2152	1.3934
1998	6.5804	1.573	6.2973	1.4046
1999	6.3472	1.7145	6.3793	1.4159
2000	6.7535	1.5164	6.4614	1.4272
2001	6.5388	1.7943	6.5434	1.4384
2002	6.9373	1.3865	6.6254	1.4497
2003	6.4961	1.1416	6.7075	1.4609
2004	7.3667	1.16	6.7895	1.4722
2005	7.2916	1.0217	6.8716	1.4834
2006	6.7774	1.6811	6.9536	1.4947
2007	7.2126	1.4774	7.0357	1.5060
2008	7.399	1.4451	7.1177	1.5172
2009	7.2871	1.6959	7.1997	1.5285
2010	6.9299	1.6984	7.2818	1.5397
2011	7.2031	1.4834	7.3638	1.5510
2012	7.3214	1.3336	7.4459	1.5622
2013	7.4551	1.6739	7.5279	1.5735
2014	7.5153	1.9154	7.6099	1.5848
2015	7.0112	1.3706	7.6920	1.5960
2016	7.4819	1.7396	7.7740	1.6073
2017	7.6552	1.5911	7.8561	1.6185

Table 2 shows the relative long term rises for all ages by ethnicity and the applicable periods for which they have been monitored. Only nine cancer registries contribute to long term cancer data in the SEER database. More recent data 2001–2017 is contributed by 21 registries.

**Table 2:** Long Term Testicular Cancer Incidence Trends by Ethnicity

Ethnicity	Start Year	End Year	Initial Rate	End Rate	Change	Rise
***Observed Rates***						
NH_Caucasian-American	2001	2017	5.0632	5.0834	0.0203	0.40%
Hispanic-American	2003	2016	3.3823	4.6036	1.2213	36.11%
NH_African-American	2002	2017	1.1420	1.1571	0.0151	1.32%
Asian-Pacific Islander-American	2003	2017	0.9368	1.9098	0.9730	103.87%
***Modelled Rates***						
NH_African-American	1975	2017	1.1457	1.6185	0.4728	41.27%
NH_Caucasian-American	1975	2017	4.4103	7.8561	3.4457	78.13%
Relative Elevation of Rates			3.8495	4.8539	7.2879	1.8932

Table 2 is notable for marked ethnic disparities in the rate of rise of the TCR. For example across the period cited the Non-Hispanic Caucasian American population TCR grew only 0.4% compared to the growth in the Hispanic-American TCR of 36.11% and in the Asian-Pacific Islander -American population where it grew 103.87% albeit from a lower starting point. This may imply differing exposures to some environmental intoxicant.

The long term data appears at the bottom of this table. One notes that the growth of the TCR in the African-American community of 41.27% was only 52.82% of the growth of the Caucasian-American community which was 78.13%.

**Fig. 9:**
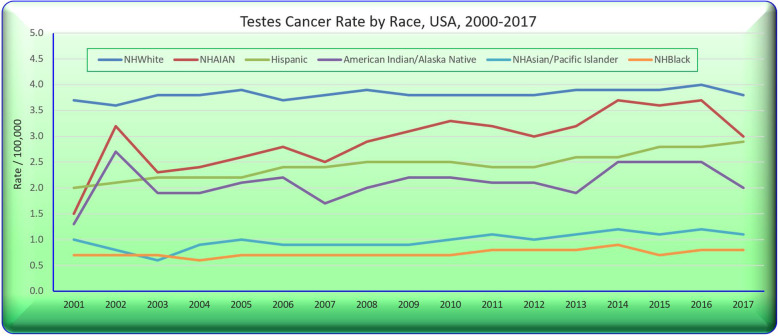
Testicular cancer rates by selected ethnicities

Figure 9 sets out these trends by ethnicity across all ages.

**Fig. 10:**
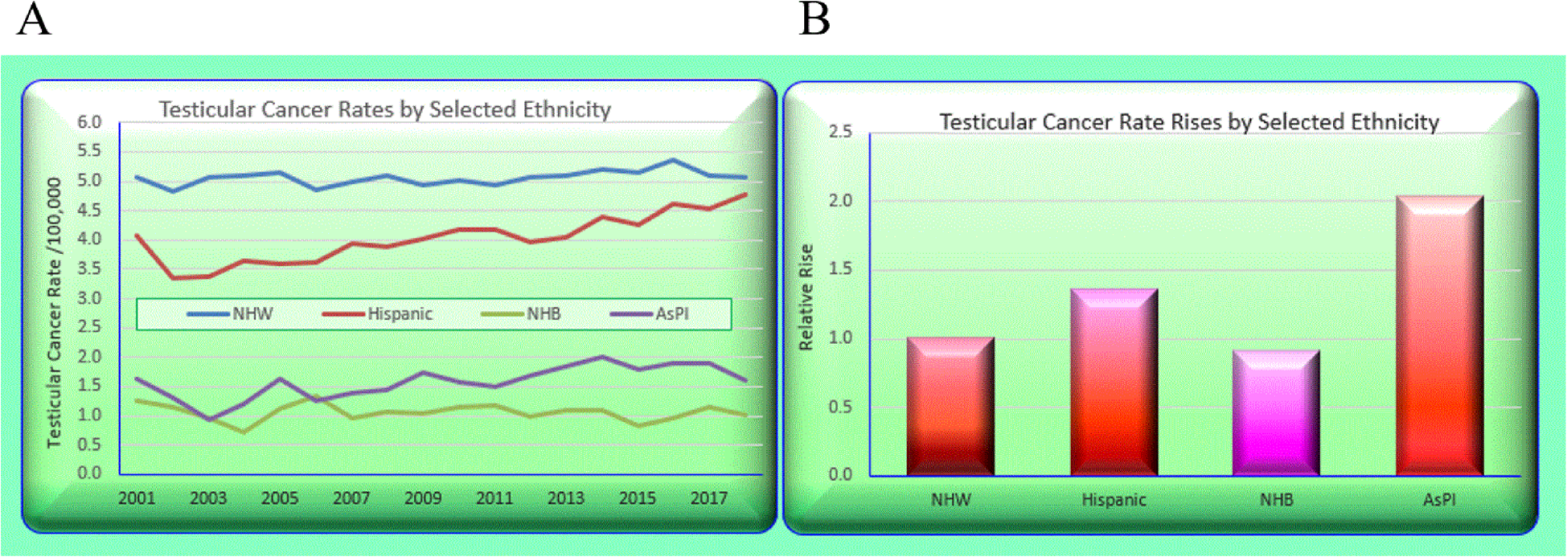
Testicular cancer rates derived from state data by selected ethnicity. (**A**) Over time. (**B**) Rises across periods

Figure 10A again sets out the ethnic trends and the bar chart in Panel B shows the relative rises in each of the four groups.

**Fig. 11:**
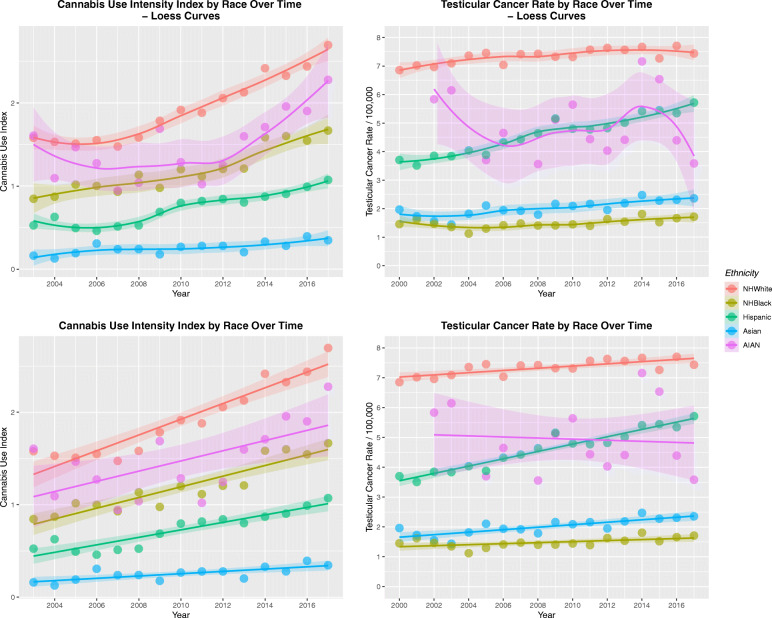
Cannabis use and testicular cancer rates by time and by aggregate time. (**A** and **C**) cannabis use, (**B** and **D**) testicular cancer rates. (**A** and **B**) loess curves of best fit. (**C** and **D**) Least squares regression lines

Figure 11 shows four scatterplots of the cannabis use intensity index by ethnicity (Panels A and C) and the TCRs by ethnicity (Panels B and D) as loess curves (A, B) and regression lines of best fit (C, D). One notes a very striking resemblance between the two sets of graphs.

**Fig. 12:**
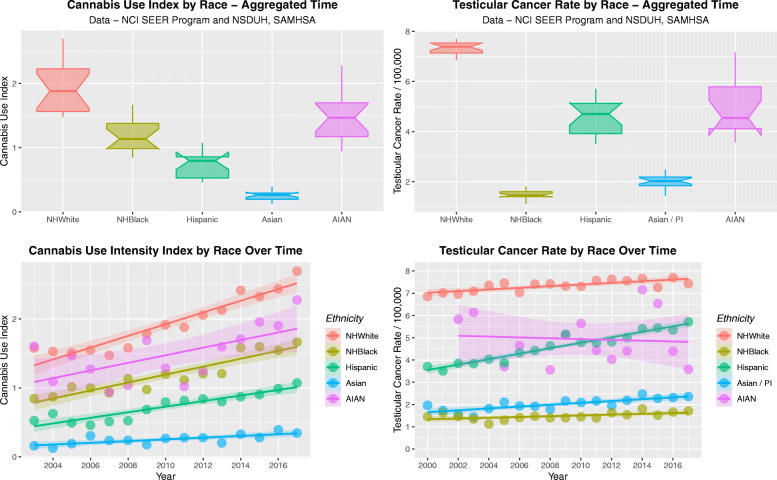
Cannabis use and testicular cancer rates by time and by aggregate time. (**A** and **C**) cannabis use, (**B** and **D**) testicular cancer rates. (**A** and **B**) Boxplots; (**C** and **D**) Regression lines

Figure 12 sets out these data as boxplots in panels A and B and time-dependent regression lines in panels C and D. One reads the boxplot graphs by noting that groups which do not have overlapping notches are statistically significantly different from each other. The broad parallels between the two sets of plots is again apparent.

**Fig. 13:**
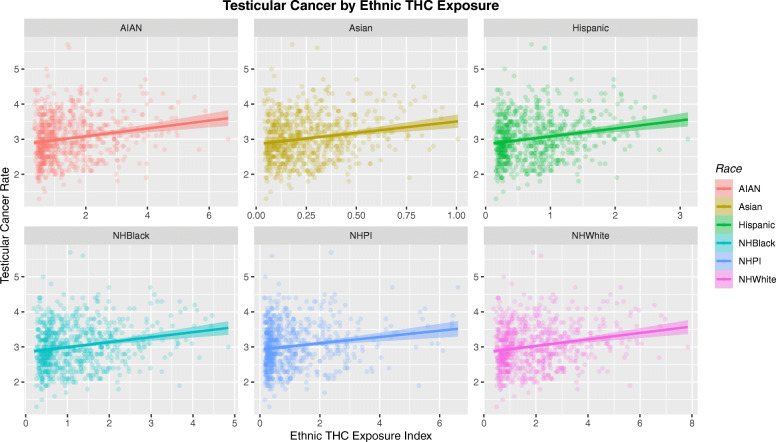
Testicular cancer rates by ethnic exposure to THC. Note variable scales on horizontal-axis

Figure 13 sets out the relationship of the TCR to the ethnic THC exposure index by ethnicity. Whilst each of these six plots look similar careful comparison shows that the scales on the horizontal axis are very different.

**Fig. 14:**
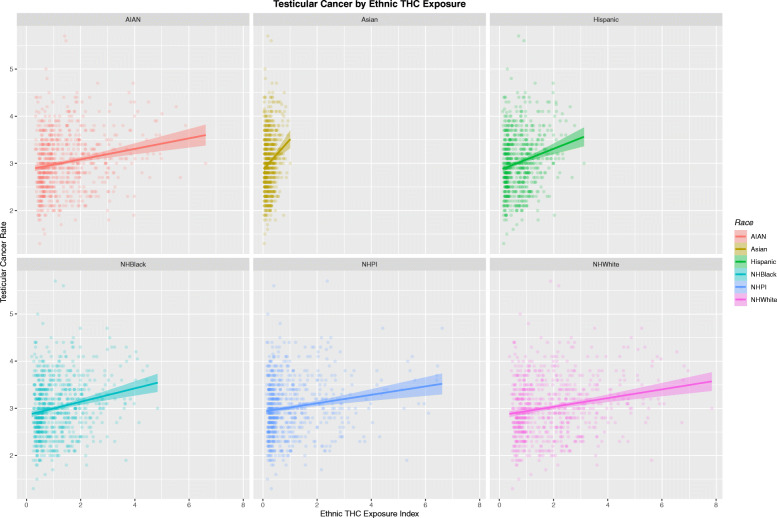
Testicular cancer rates by ethnic exposure to THC. Note fixed scales on horizontal-axis and variable slope of regression lines

These data are therefore re-plotted with comparable axis scales in Fig. 14. Now two striking trends appear. Firstly the much higher THC exposure in the Caucasian-American and AIAN-American groups is very apparent. Also the slope of the regression lines in each case is very different. Hence the regression line for the Asian-American group is very short in horizontal scope, but very steep. This graph is very thought-provoking and has far reaching implications indeed. These differences clearly indicate major impacts of cannabis exposure by ethnic background and may potentially be related to one or several genomic or epigenomic processes acting in concert.

**Fig. 15:**
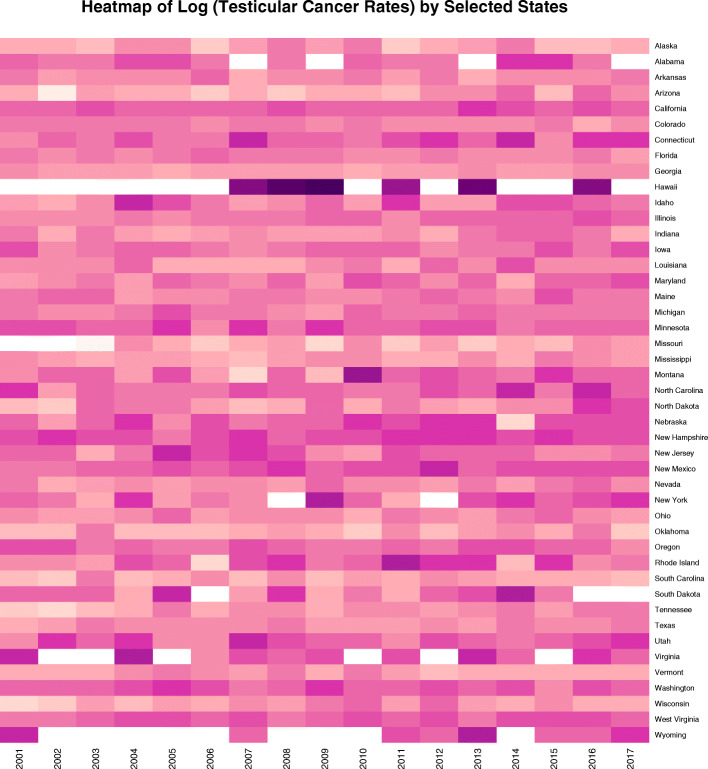
Heatmap of Caucasian-American testicular cancer incidence rates by state and year. Note very high rate for Hawaii near the top

Although this paper focusses on the ethnic disparities it should be pointed out that each ethnicity is not one homogenous block. The heatmap in Fig. 15 shows the log (TCR) in the Caucasian-American community and notes an obvious hotspot in Hawaii which has rates in each of the nominated years of 9.4, 11.2, 12.7, 8.8, 10.4 and 9.4 (10.32 + 0.59 mean + S.E.M.) which is much higher than the comparable rates for the Caucasian-American ethnicity in all other states which is 5.351 + 0.033 (t = 115.12, df = 738.37, *P* = 0.0000; for comparison t = 67, df = 738.37, *P* = 2.44 × 10^− 316^).

We now turn to the formal analysis of the state-based data for ages 15–60 years derived from the most recent datafiles held in the National Program of Cancer Registries and the SEER Incidence – US Cancer Statistics Public Use Database 2019 submission 2001–2017 which includes data for the whole country.

Inverse probability weights may be calculated for this data which control for the effect of cannabis exposure as a function of the other substance exposures.

Table 3 presents the results of linear regressions of the TCR against ethnicity and the ethnic THC exposure in both wide and long datasets. In the latter all the ethnicities are collated into one column and the data-table becomes longer by a factor of the number of ethnicities.

**Table 3:** Introductory Linear Regressions

Parameter Estimates			Model Parameters				
Parameter	Estimate (C.I.)	***P***-Value	S.D.	R-Squared	F	dF	P
***Race***							
***(lm(Rate ~ Race)***							
Caucasian-American	4.6441 (4.2, 5.08)	8.30E-31	0.8828	0.8657	426.3	1,65	2.98E-30
(lm(Rate ~ Year * Race)							
Caucasian-American	− 137 (− 175.61, − 98.39)	2.47E-09	0.3069	0.9838	1334	3,63	6.27E-57
Year: Caucasian-American	0.08 (0.07, 0.09)	< 2.2E-16					
lm(TC_Rate ~ EthnTHCExp * Race)							
log(EthnTHCExp)	0.06 (0.04, 0.08)	1.17E-07	0.2017	0.0293	13.34	11,4488	< 2E-16
RaceAsian	0.09 (0.05, 0.12)	1.46E-05					
RaceHispanic	0.04 (0.01, 0.06)	0.0021					
RaceNHPI	0.02 (0, 0.04)	0.0456					
***In Ethnic Dataset, Long***							
***(lm(Rate ~ Cannabis)***							
Cannabis	0.47 (0.41, 0.52)	1.70E-69	0.5922	0.0663	320.2	14,498	3.10E-69
lm(Rate ~ Time * Cannabis)							
Cannabis	0.49 (0.44, 0.55)	9.89E-64	0.5919	0.0671	162.6	24,497	1.31E-36
Year	−0.0049 (− 0.0093, − 0.0004)	0.03169					

In each case ethnicity and the ethnic THC exposure is noted to be a highly significant covariate of TCR.

### Multivariable adjustment

Table 4 presents the results of inverse probability weighted mixed effects regression all from the long dataset. Many terms involving ethnicity and ethnic THC exposure are highly significant. In an additive model with the other four drugs ethnic THC exposure is significant (β-estimate = 0.05 (0.04, 0.06), *P* = 5.80 × 10^− 31^). In a four-way interactive model with other drugs ethnic cannabis exposure is significant (β-estimate = 0.64 (0.17, 1.10), *P* = 0.0072).

**Table 4:** Mixed Effects Model Regressions

Parameters			Model Parameters			
Parameter	Estimate (C.I.)	*P*-Value	SD	AIC	BIC	logLik
***lme(TestCa ~ Race)***						
Asian-Am.	− 0.2 (− 0.39, − 0.02)	0.0320	1.141	3566.977	3595.930	− 1777.489
Hispanic-Am.	2.82 (2.65, 3)	2.5E-150				
NHCaucasian-Am.	4.17 (4, 4.34)	1.3E-246				
***lme(TestCa ~ Ethnic_THC_Exposure)***						
Ethnic_THC_Exp	0.05 (0.04, 0.06)	4.40E-31	0.202	− 1595.682	−1570.036	801.841
***lme(TestCa ~ Race + Ethnic_THC_Exposure)***						
NHCaucasian-Am.	4.06 (3.88, 4.23)	1.13E-234	1.126	3546.406	3580.177	− 1766.203
Hispanic-Am.	2.93 (2.76, 3.11)	4.23E-155				
Ethnic_THC_Exp	0.24 (0.15, 0.32)	2.13E-07				
lme(TestCa ~ Race * Ethnic_THC_Exposure)						
NHCaucasian-Am.	4.12 (3.95, 4.3)	9.4E-236	1.115	3539.109	3587.320	− 1759.554
Hispanic-Am.	3.17 (2.96, 3.37)	1.5E-141				
Hispanic-Am.: Ethnic_THC_Exp	0.58 (0.4, 0.77)	6.6E-10				
NHCaucasian-Am.: Ethnic_THC_Exp	0.16 (0.05, 0.27)	0.0044				
Asian-Am.: Ethnic_THC_Exp	0.15 (−0.12, 0.43)	0.2753				
Asian-Am.	0.06 (− 0.42, 0.54)	0.8075				
NHAfrican-Am.: Ethnic_THC_Exp	−0.05 (− 0.35, 0.25)	0.7381				
lme(TestCa ~ Year * Race)						
Year: Hispanic-Am.	0.2 (0.17, 0.22)	2.9E-38	1.036	3427.797	3476.008	− 1703.898
Year: NHCaucasian-Am.	0.02 (0.01, 0.04)	0.0032				
Hispanic-Am.	−396.69 (− 504.97, −288.41)	1.5E-12				
lme(TestCa ~ Year * Ethnic_THC_Exposure)						
Ethnic_THC_Exp	1.39 (1.25, 1.53)	1.51E-72	2.110	4659.713	4683.846	− 2324.857
Year	−0.14 (−0.18, − 0.11)	2.21E-17				
***Additive Model***						
***lme(Rate ~ Cigarettes + AUD + Ethnic_THC_Exp + Analgesics + Cocaine)***						
AUD	5.49 (5.11, 5.87)	6.57E-162	0.180	− 2610.768	− 2559.484	1313.384
Ethnic_THC_Exp	0.05 (0.04, 0.06)	5.80E-31				
Cocaine	−2.46 (−3.3, −1.62)	1.17E-08				
Analgesics	−0.13 (− 0.16, − 0.1)	3.09E-21				
Cigarettes	− 1.12 (− 1.27, − 0.97)	7.42E-49				
***Interactive Model - 3-Way***						
lme(Rate ~ Cigarettes * AUD * Ethnic_THC_Exp + Analgesics + Cocaine)						
Cigarettes: AUD	23.11 (21.54, 24.67)	2.14E-169	0.179	− 2675.013	− 2617.321	1346.507
Cigarettes: Ethnic_THC_Exp	0.44 (0.31, 0.57)	5.22E-11				
Ethnic_THC_Exp	−0.05 (− 0.08, − 0.01)	3.80E-03				
Cocaine	−1.96 (−2.8, − 1.11)	6.09E-06				
Analgesics	− 0.13 (− 0.16, − 0.11)	2.56E-22				
Cigarettes	− 2.4 (− 2.59, − 2.2)	2.39E-124				
***Interactive Model - 4-Way***						
lme(Rate ~ Cigarettes * AUD * Ethnic_THC_Exp * Analgesics + Cocaine)						
Cigarettes: AUD	23.48 (21.9, 25.05)	6.73E-172	0.177	− 2725.041	− 2648.125	1374.520
Analgesics	0.31 (0.15, 0.48)	0.0002				
Ethnic_THC_Exp: Analgesics	0.22 (0.07, 0.37)	0.0036				
Ethnic_THC_Exp	0.64 (0.17, 1.1)	0.0072				
Cigarettes: Ethnic_THC_Exp	−3.8 (−5.8, −1.79)	0.0002				
Cigarettes: Ethnic_THC_Exp: Analgesics	−1.38 (−2.03, −0.73)	3.19E-05				
Cocaine	−1.87 (−2.71, − 1.02)	1.62E-05				
Cigarettes: Analgesics	−2.21 (− 2.96, − 1.47)	6.05E-09				
Cigarettes	−9.3 (− 11.61, −6.99)	3.98E-15				

Table 5 presents the results of an inverse probability weighted two-step instrumental variable regression model on the long dataset. The table is very interesting. When race alone is considered in the first model with African-American race as the comparator, no significant changes are seen. However when ethnic THC exposure is considered it is very highly significant. When race and ethnic THC exposure are considered in an additive model all parameters are significant and ethnic THC exposure is the most significant.

**Table 5:** Mixed Effects Model Regressions

Parameters				Model Parameters				
Instrumental Variable	Parameter	Estimate (C.I.)	*P*-Value	S.D.	R-Squared	Wald ChiSqu.	dF	P
	***ivreg(TestCa ~ Race)***							
	Race_Asian	0.26 (−25.24, 25.77)	0.984	132.073	0.8888	2463	3921	< 2.2E-16
	Race_Hispanic	2.7 (−13.73, 19.12)	0.748					
	Race_NHWhite	3.5 (−12.93, 19.92)	0.677					
	***ivreg(TestCa ~ Ethnic_THC_Exposure)***							
	Ethnic_THC_Exp	0.89 (0.88, 0.91)	<< 2.3E-320	102.3782	0.9332	1.29E+ 04	1923	<< 2.3E-320
	***ivreg(Rate ~ Ethn_THC_Exposure + Race)***							
	Ethn_THC_Exposure: NHCaucasian-Am._THC_Exposure	0.08 (0.02, 0.13)	0.0095	8.4472	0.0361	29.06	64,493	<< 1.0E-320
	Ethn_THC_Exposure: NHAfrican-Am._THC_Exposure	−0.09 (−0.14, −0.03)	0.0032					
	Ethn_THC_Exposure: NHPI_THC_Exposure	−0.19 (− 0.25, − 0.13)	3.4E-09					
	Ethn_THC_Exposure: Hispanic-Am._THC_Exposure	−0.25 (− 0.32, − 0.18)	4.2E-13					
	Ethn_THC_Exposure: Asian-Am._THC_Exposure	− 0.63 (− 0.74, − 0.52)	3.6E-29					
	Ethnic_THC_Exp	− 0.35 (− 0.4, − 0.3)	1.5E-38					
	ivreg(TestCaRt ~ log(EthnTHCExp) * Race)							
	NHWhite: Ethn_THC_Exp	1.22 (1.16, 1.27)	6.9E−128	95.9894	0.9430	3703	4920	< 2e-16
	Hispanic: Ethn_THC_Exp	1.48 (1.38, 1.59)	5.6E-214					
	ivreg(TestCaRt ~ log(EthnTHCExp) * Race)							
THC	RaceAsian	3.88E+ 05 (−8.93E+ 07, 9.01E+ 07)	0.993	130,195	-1.1E-05	9.013E+ 05	3921	1.0000
Cannabigerol	RaceHispanic	1.47E+ 05 (−3.38E+ 07, 3.40E+ 07)	0.993					
Cannabinol	RaceNHWhite	1.47E+ 05 (− 3.38E+ 07, 3.41E+ 07)	0.993					
	ivreg(TestCa ~ Cigarettes * AUD * Ethn_THC_Exposure + Race + Cocaine + MHY + Analgesics)							
	AUD	138.37 (126.96, 149.78)	< 2e-16	24.2846	0.9962	2.04E+ 04	12,912	< 2.2E-16
	AUD: Ethnic_THC_Exp	312.53 (290.54, 334.53)	< 2e-16					
	Cigarettes: Ethnic_THC_Exp	150.41 (139.67, 161.15)	< 2e-16					
	Analgesics	1.07 (0.67, 1.47)	1.8E-07					
	NHCaucasian-Am.	3.25 (0.22, 6.28)	0.0361					
	Hispanic-Am.	2.91 (−0.12, 5.93)	0.0597					
	Cocaine	− 132.56 (−150.11, −115.01)	< 2e-16					
	Cigarettes	−45.52 (−50.67, −40.37)	< 2e-16					
	Ethnic_THC_Exp	−22.08 (−24, −20.17)	< 2e-16					
	MHY	−12.03 (− 12.98, − 11.08)	< 2e-16					
	Cigarettes: AUD: Ethnic_THC_Exp	− 2032.86 (− 2169.16, − 1896.55)	< 2e-16					
	ivreg(TestCa ~ Cigarettes * AUD * Ethnic_THC_Exp * Race + Cocaine + MHY + Analgesics)							
THC	Cigarettes	30.94 (21.36, 40.52)	3.9E-10	16.2755	0.0138	20.04	3921	1.37E-12
Cannabigerol	Ethnic_THC_Exp	0.55 (0.05, 1.05)	0.0298					
Cannabinol	AUD	−49.84 (−64.89, −34.78)	1.4E-10					
	ivreg(Rate ~ Cigarettes * AUD + Analgesics + Cocaine + MHY + Race * Ethnic_THC_Exposure)							
	Cocaine	61.73 (56.9, 66.56)	6.1E-130	6.8060	0.3801	173.4	16,4483	<< 1.0E-320
	Cigarettes: AUD	142.1 (126.47, 157.73)	5.2E-69					
	NHPI	0.52 (0.34, 0.7)	1.8E-08					
	Ethnic_THC_Exp	0.32 (0.2, 0.43)	4.7E-08					
	Asian-Am.	0.44 (0.25, 0.63)	6.8E-06					
	Hispanic-Am.	0.24 (0.06, 0.42)	0.00752					
	Asian-Am.: Ethnic_THC_Exp	−0.41 (−0.56, −0.26)	1.68E-07					
	NHPI: Ethnic_THC_Exp	−0.37 (− 0.5, − 0.24)	2.96E-08					
	Analgesics	−1.13 (−1.29, − 0.97)	7.65E-44					
	Cigarettes	−38.49 (−42.35, −34.63)	3.90E-82					
	MHY	−9.58 (−10.06, −9.1)	4.1E-289					

When Race and ethnic THC exposure are considered in an interactive model Caucasian-American and Hispanic-American races both in interaction with ethnic THC exposure are highly significant (β-estimate = 1.48 (1.38, 1.59), *P* = 5.62 × 10^− 214^ and β-estimate = 1.22 (1.16, 1.27), *P* = 65.86 × 10^− 128^ respectively).

However, when the same regression is performed with THC, cannabigerol and cannabinol as instrumental variables significance amongst both the terms and the model is lost and adjusted R-squared falls from 0.9430 to − 0.0000108.

Similarly when all substances and income are considered along with ethnic THC exposure, two interactive terms including ethnic THC exposure are positive and highly significant.

However when THC, cannabigerol and cannabinol are included as instrumental variables again there is a marked collapse of significant findings, adjusted R-squared falls from 0.9962 (which is very high) to 0.0138 (quite low) and the model Wald coefficient falls from 20,000 to 20.04.

In the final model in this table using a different interaction structure the ethnic THC exposure is again independently highly statistically significant (β-estimate = 0.32 (0.20, 0.43), *P* = 4.7 × 10^− 8^).

Table 6 presents the results of inverse probability weighted robust regressions in the long dataset. Ethnic THC exposure is clearly highly significantly prominent. As model complexity increases so the significance of terms including the cannabinoids increases.

**Table 6:** Robust Inverse Probability Weighted Regression

Parameter	Estimate (C.I.)	*P*-Value
***Additive Model with State Cannabis***		
***svyglm(TestCaRt ~ Cigarettes + Cannabis + + AUD + Analgesics + Cocaine)***		
AUD	64.55 (56.89, 72.22)	< 2.2E-16
Analgesics	11.01 (6.24, 15.77)	5.7E-05
NHWhite	5.44 (2.36, 8.53)	0.0013
Hispanic	4.63 (1.54, 7.71)	0.0055
Cannabis	−9.38 (−12.76, −5.99)	3.4E-06
Cocaine	−131.14 (− 138.96, − 123.33)	< 2.2E-16
***Additive Model with Ethnic THC Exposure***		
svyglm(TestCaRt ~ Cigarettes * Ethn_THC_Exposure * + AUD + Analgesics + Cocaine)		
Cigarettes	28.96 (27.87, 30.05)	< 2E-16
Hispanic	2.55 (2.05, 3.04)	1.8E-12
Asian	4.89 (1.08, 8.69)	0.0160
Ethn_THC_Exposure	2.9 (0.41, 5.38)	0.0281
Cocaine	−56.03 (−110.14, − 1.93)	0.0492
***Interactive Model with Ethnic_THC_Exposure***		
svyglm(TestCaRt ~ Cigarettes * Ethn_THC_Exposure * + AUD + Analgesics + Cocaine + Race + Income)		
Ethn_THC_Exposure	4.72 (2.04, 7.41)	0.0018
Asian	3.93 (1.64, 6.22)	0.0022
Cigarettes	11.09 (3.77, 18.42)	0.0060
Cigarettes: NHWhite	109.87 (24.24, 195.5)	0.0177
Cigarettes: Ethn_THC_Exposure: Asian	17.32 (1.64, 33.01)	0.0388
NHWhite	−19.53 (−39.11, 0.05)	0.0603
Ethn_THC_Exposure: Asian	−4.45 (−8.41, −0.5)	0.0352
Cocaine	−88.11 (−161.51, − 14.7)	0.0257
Cigarettes: Ethn_THC_Exposure	−20.11 (− 32.44, −7.78)	0.0034
Cigarettes: Asian	−16.35 (−26.11, −6.6)	0.0027
***Interactive Model with State Cannabis***		
svyglm(TestCaRt ~ Cigarettes * Cannabis * + AUD + Analgesics + Cocaine + Race + Income)		
AUD	13.58 (7.69, 19.47)	0.0001
Cigarettes: Cannabis: NHWhite	398.68 (214.58, 582.79)	0.0002
Cigarettes: NHWhite	933.24 (493.05, 1373.44)	0.0003
Cigarettes: Hispanic	1372.45 (672.78, 2072.11)	0.0007
Cigarettes: Cannabis: Hispanic	635.8 (305.36, 966.24)	0.0008
Analgesics	19.88 (9.19, 30.57)	0.0011
Cannabis	42.63 (18.65, 66.61)	0.0017
Cigarettes: Cannabis	−199.68 (− 315.91, −83.44)	0.0023
Cigarettes	− 578.67 (− 907.11, − 250.23)	0.0018
Cocaine	−75.51 (− 117.23, −33.78)	0.0015
Cannabis: Hispanic	− 144.06 (− 218.53, −69.59)	0.0008
Hispanic	− 308.36 (− 466.63, − 150.09)	0.0007
NHWhite	−214 (− 315.14, − 112.87)	0.0003
Cannabis: NHWhite	−93.06 (− 135.37, −50.75)	0.0002
***Interactive Model with State Cannabinoids***		
svyglm(TestCaRt ~ Cigarettes * THC * Cannabigerol * + AUD + Analgesics + Cocaine + Race + Income)		
Cigarettes: NHWhite	127.24 (92.37, 162.1)	4.7E-07
Cigarettes: THC: Cannabigerol: NHBlack	13.87 (6.33, 21.41)	0.0017
Cigarettes: NHBlack	53.77 (18.14, 89.41)	0.0075
Cigarettes: THC: NHBlack	49.95 (14.93, 84.96)	0.0108
Cigarettes: Cannabigerol: NHBlack	14.62 (3.67, 25.57)	0.0161
Cigarettes: THC: Cannabigerol: NHWhite	35.97 (7.97, 63.97)	0.0200
Cigarettes: THC: Hispanic	214.68 (31.83, 397.53)	0.0317
Cigarettes: Hispanic	−310.38 (− 540.45, −80.31)	0.0152
Cigarettes: Cannabigerol: Hispanic	−89.37 (−155.46, −23.28)	0.0150
AUD	−43.07 (−68.76, −17.38)	0.0035
THC: Cannabigerol: NHWhite	−8.18 (−11.39, − 4.96)	6.2E-05
NHWhite	−16.98 (−23.16, − 10.81)	2.4E-05

### Multiple imputation of missing ethnicity data

It would be useful to study these ethnic effects in further detail. However if one simply considers the Caucasian-American and African-American datasets it is noted that 1782 of 2700 datapoints are absent, or 66.0%. This is a severe limitation on further detailed analysis.

For this reason formal data imputation by multiple imputation of chained equations was performed using the R-package mice [38].

Table 7 shows the impact of missing data by ethnicity. Mean and median data and missing data rates are indicated.

**Table 7:** Missing Data Considerations

Ethnicity	Mean Rate Overall	Median Rate Overall	Mean Rate from Population Totals	Mean Rate from Population Rates
NHWhite	5.37	5.40	5.39	5.39
NHBlack	**1.09**	**1.00**	1.23	1.24
Hispanic	**3.31**	**3.35**	3.63	3.70
Asian-PI	**1.26**	**1.20**	1.92	2.03
**Ethnicity**	**Observations**	**Total**	**Missing Values**	**% Missing**
NHWhite	644	675	31	**4.59%**
NHBlack	63	675	612	**90.67%**
Hispanic	177	675	498	**73.78%**
Asian-PI	34	675	641	**94.96%**

**Fig. 16:**
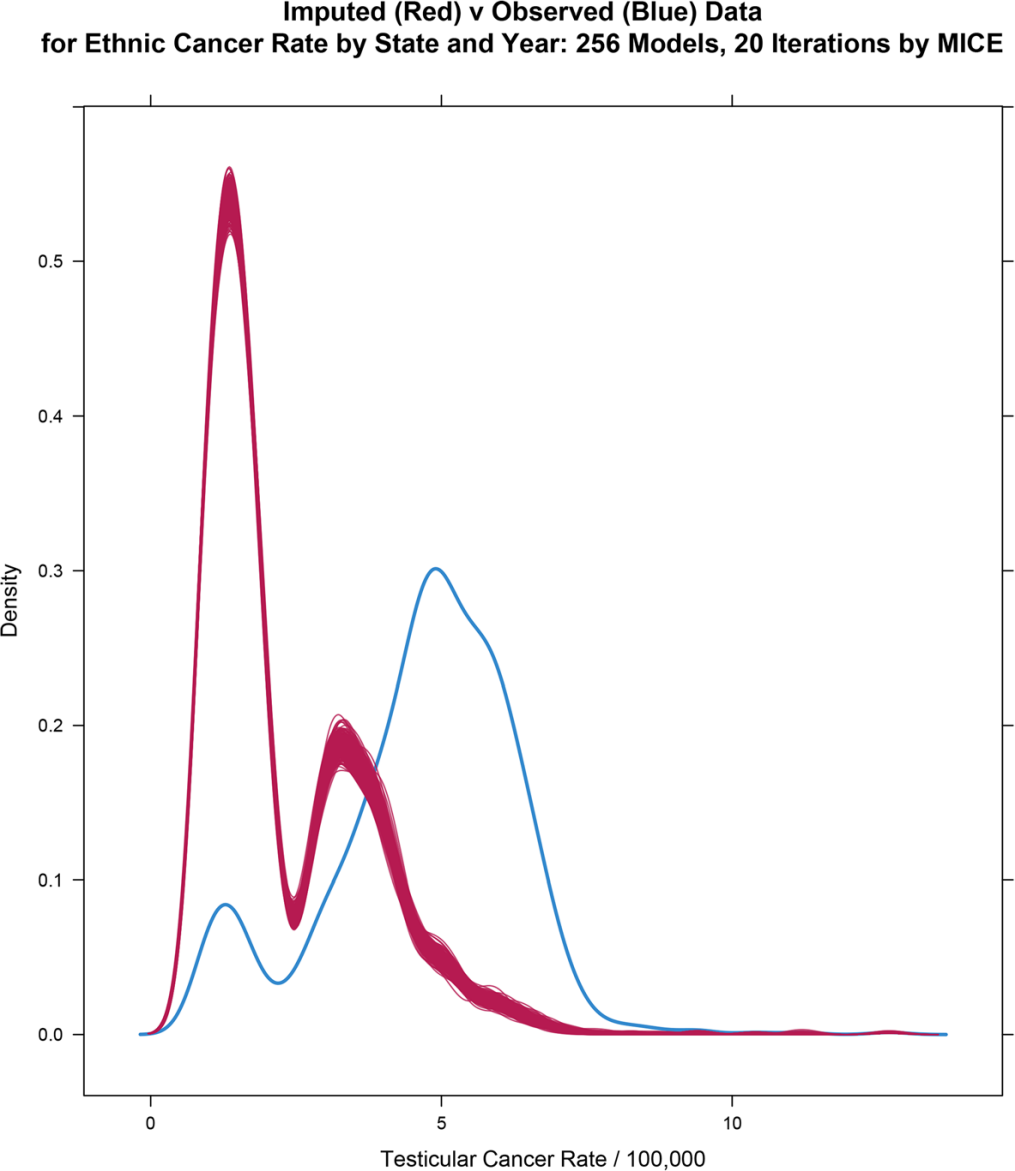
Density plot for imputed values for ethnicity data. Note lower peaks for ethnic minority rates

Following [40] 256 imputations were performed with 20 iterations each. Figure 16 shows the density plot of the multiply imputed data. One notes the obvious peaks in the lower areas corresponding to the smaller values of the ethnic minorities.

**Fig. 17:**
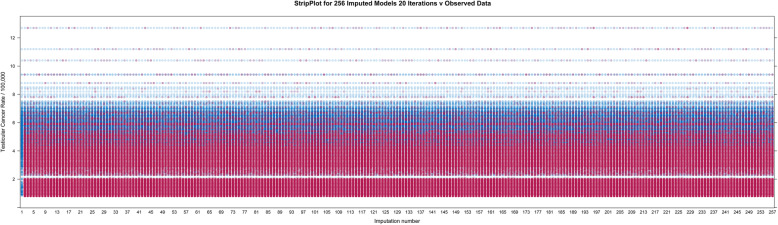
Stripplot for multiply imputed data. Note that the imputed values are all taken from the values of the available data

Figure 17 is a strip plot illustrating how the imputed values nicely follow the values of the known observed data including their distribution pattern.

**Fig. 18:**
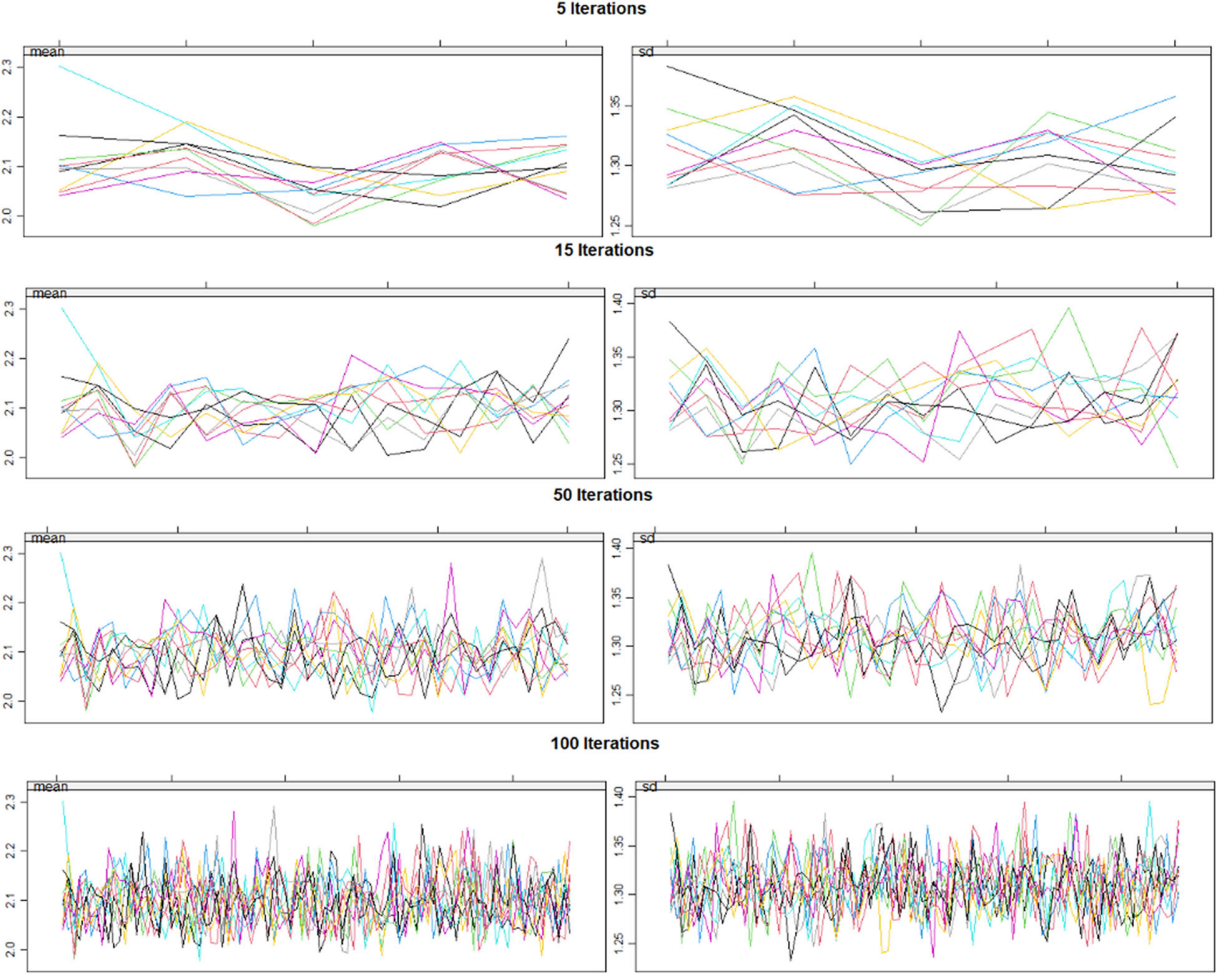
Imputation convergence. Note increasing convergence of data with increasing iterations of imputation algorithm

Figure 18 demonstrates the manner in which the first 100 imputations nicely converge.

Multiple imputation in this manner allows the calculation of various regression equations from the data with pooling of the final models into a meaningful outcome by applying Rubin’s rules for pooling of such chained models.

Table 8 shows the result of various linear model formulae performed in this way on the imputed long dataset. Ethnic cannabis exposure is noted to continue to be highly significant. Quintile effects are also demonstrated.

**Table 8:** Linear Regressions on Multiply Imputed Data

Parameters			Model			
Parameter	Estimate (C.I.)	*P*-Value	No. Imputa- tions	S.D.	Lambda	FMI
***Ethnic_THC_Exposure Alone***						
***lm(TestCaRt ~ Ethnic_THC_Exposure)***						
Ethnic_THC_Exposure	0.68 (0.62, 0.74)	1.80E-92	256	1.7000	0.0292	0.0299
Ethnic_THC_Exposure * Race						
***lm(TestCaRt ~ Ethnic_THC_Exposure + Race)***						
NHWhite-American	4.17 (4.08, 4.26)	<< 9.8E-324	256	0.8183	0.0560	0.0568
Hispanic-American	2.63 (2.53, 2.73)	<< 2.4E-315			0.1681	0.1690
Asian-American	1.1 (0.98, 1.21)	2.45E-70			0.1018	0.1026
Ethnic_THC_Exposure	0.19 (0.14, 0.24)	7.99E-15			0.1521	0.1530
***Additive - Drugs***						
***lm(TestCaRt ~ Cigarettes + AUD + Ethnic_THC_Exposure + Analgesics + Cocaine)***						
Ethnic_THC_Exposure	0.72 (0.66, 0.79)	9.10E-99	256	1.6893	0.0313	0.0321
AUD	12.64 (8.37, 16.91)	7.28E-09			0.0391	0.0399
***Interactive Full Model***						
***lm(TestCaRt ~ Cigarettes + AUD + Ethnic_THC_Exposure + Analgesics + Cocaine + 5_Races + MHY + Cigarettes.AUD + Cigarettes.EthnTHCExpRt + EthnTHCExpRt.AUD)***						
NHWhite-American	4.2 (4.11, 4.29)	<< 3.1E-322	256	0.8017	0.0636	0.0644
Hispanic-American	2.59 (2.5, 2.69)	<< 8.9E-323			0.1727	0.1737
Asian-American	0.99 (0.87, 1.11)	1.99E-52			0.1129	0.1138
AUD	6.45 (4.21, 8.68)	2.01E-08			0.1491	0.1500
Ethnic_THC_Exposure	0.12 (0.07, 0.18)	1.89E-05			0.1658	0.1667
Analgesics	−5.31 (−8.82, −1.8)	3.04E-03			0.1167	0.1175
Cigarettes	−3.72 (−4.64, −2.8)	3.55E-15			0.1336	0.1345
***Quintiles***						
***lm(TestCaRt ~ Quintile)***						
Quintile 2	0.02 (−0.2, 0.24)	0.8801	256	1.8379	0.0261	0.0268
Quintile 3	0.1 (−0.12, 0.32)	0.3566			0.0295	0.0302
Quintile 4	0.2 (−0.02, 0.42)	0.0755			0.0247	0.0255
Quintile 5	0.29 (0.06, 0.51)	0.0118			0.0331	0.0338
***Dichotomized Quintiles***						
***lm(TestCaRt ~ Dichotomized_Quintiles)***						
Upper_2_Quintiles	0.2 (0.06, 0.35)	0.0058	256	1.8375	0.0309	0.0316

### Causal inference

Table 9 sets out the e-Values relating to the various calculations presented above. Minimum e-Values are set out in Table 10 listed in descending order. One notes that 29/37 e-Values are > 1.25 which is said to be the cut-off for causal attribution [41].

**Table 9:** e-Values

Parameter	Estimate (C.I.)	R.R. (C.I.)	E-Values
***LINEAR MODELS***			
lm(TC_Rate ~ EthnTHCExp * Race)			
Ethnic_THC_Exposure	0.06 (0.04, 0.08)	1.31 (1.18, 1.45)	1.95, 1.66
***In Ethnic Dataset, Long***			
***(lm(Rate ~ Race)***			
Cannabis	0.47 (0.41, 0.52)	2.04 (1.68, 2.47)	3.05, 2.76
lm(Rate ~ Time * mrjmon)			
Cannabis	0.49 (0.44, 0.55)	2.19 (1.95, 2.32)	3.68, 3.31
***MIXED EFFECTS MODELS***			
***lme(TestCa ~ Ethnic_THC_Exposure)***			
Ethnic_THC_Exp	0.05 (0.04, 0.06)	1.23 (1.19, 1.28)	1.78, 1.68
***lme(TestCa ~ Race + Ethnic_THC_Exposure)***			
Ethnic_THC_Exp	0.24 (0.15, 0.32)	1.21 (1.13, 1.30)	1.72, 1.50
lme(TestCa ~ Race * Ethnic_THC_Exposure)			
Hispanic-Am.: Ethnic_THC_Exp	0.58 (0.4, 0.77)	1.67 (1.27, 2.26)	2.74, 1.85
lme(TestCa ~ Year * Ethnic_THC_Exposure)			
Ethnic_THC_Exp	1.39 (1.25, 1.53)	1.82 (1.72, 1.93)	3.05, 2.83
***Additive Model - Drugs***			
Ethnic_THC_Exp	0.05 (0.04, 0.06)	1.29 (1.23, 1.34)	1.89, 1.77
***Interactive Model - 3-Way***			
Cigarettes: Ethnic_THC_Exp	0.44 (0.31, 0.57)	949 (4.86, 18.54)	18.8, 9.20
***Interactive Model - 4-Way***			
Ethnic_THC_Exp: Analgesics	0.22 (0.07, 0.37)	3.14 (1.45, 6.79)	5.74, 2.28
Ethnic_THC_Exp	0.64 (0.17, 1.1)	26.50 (2.44, 287.17)	52.52, 4.33
***IV-REGRESSION***			
***ivreg(TestCa ~ Ethnic_THC_Exposure)***			
Ethnic_THC_Exposure	1.03 (0.89, 1.17)	1.07 (1.06, 1.07)	1.33, 1.30
***ivreg(TestCa ~ Ethnic_THC_Exposure)***			
Ethnic_THC_Exposure	0.89 (0.88, 0.91)		
***ivreg(TestCaRt ~ log(EthnTHCExp)***: ***Race)***			
Ethnic_THC_Exp: Asian-Am.	2.72 (0.84, 4.61)	1.21 (1.06, 1.38)	1.72, 1.31
Ethnic_THC_Exp: NHCaucasian-Am.	0.44 (0.3, 0.59)	1.03 (1.02, 1.04)	1.21, 1.17
ivreg(TestCa ~ Ethnic_THC_Exp * Race)			
NHCaucasian-Am.: Ethnic_THC_Exp	1.22 (1.16, 1.27)	1.01 (1.01, 1.02)	1.12, 1.12
Hispanic-Am.: Ethnic_THC_Exp	1.48 (1.38, 1.59)	1.01 (1.01, 1.02)	1.14, 1.13
ivreg(Rate ~ Cigarettes * AUD + Analgesics + Cocaine + MHY + * Ethnic_THC_Exposure)			
Ethnic_THC_Exp	0.32 (0.2, 0.43)	1.04 (1.03, 1.06)	1.25, 1.19
***ivreg(Rate ~ Ethn_THC_Exposure + Race)***			
Ethn_THC_ExposureNHCaucasian-Am._THC_Exposure	0.08 (0.02, 0.13)	1.01 (1.00, 1.01)	1.10, 1.05
ivreg(TestCa ~ Cigarettes * AUD * Cannabis + + Cocaine + MHY + Analgesics)			
AUD: Ethnic_THC_Exp	312.53 (290.54, 334.53)	1.22E+ 05 (5.36E+ 04, 2.77E+ 05)	2.43E+ 05, 1.07E+ 05
Cigarettes: Ethnic_THC_Exp	150.41 (139.67, 161.15)	280.39 (187.63, 419.03)	560.29, 374.76
ivreg(TestCa ~ Cigarettes * AUD * Ethnic_THC_Exp * + Cocaine + MHY + Analgesics)			
Ethnic_THC_Exp	0.55 (0.05, 1.05)	1.03 (1.00, 1.06)	1.21, 1.06
***MULTIPLE IMPUTATION***			
***Ethnic_THC_Exposure Alone***			
Ethnic_THC_Exposure	0.68 (0.62, 0.74)	1.44 (1.39, 1.49)	2.35, 2.13
Ethnic_THC_Exposure * Race			
Ethnic_THC_Exposure	0.19 (0.14, 0.24)	1.24 (1.17, 1.30)	1.77, 1.62
***Additive - Drugs***			
Ethnic_THC_Exposure	0.72 (0.66, 0.79)	1.48 (1.43, 1.53)	2.31, 2.20
***Interactive Full Model***			
Ethnic_THC_Exposure	0.12 (0.07, 0.18)	1.14 (1.08, 1.22)	1.56, 1.37
***Quintiles***			
Quintile 5	0.29 (0.06, 0.51)	1.15 (1.03, 1.28)	1.57, 1.21
***Dichotomized Quintiles***			
Upper_2_Quintiles	0.2 (0.06, 0.35)	1.10 (1.03, 1.18)	1.44, 1.21
***LEGAL STATUS***			
***Whites***			
Liberal	0.09 (0.06, 0.12)	1.57 (1.36, 1.81)	2.51, 2.07
***Blacks***			
Liberal	0.22 (0.12, 0.32)	2.83 (1.78, 4.84)	5.10, 2.96
***Both_Races***			
Medical	0.62 (0.43, 0.8)	1.79 (1.51, 2.13)	2.99, 2.39
Decriminalized	0.33 (0.15, 0.51)	1.36 (1.15, 1.62)	2.07, 1.56
Legal	0.61 (0.24, 0.98)	1.78 (1.25, 2.55)	2.97, 1.82
***Dichotomized_Status***			
Liberal	0.48 (0.34, 0.62)	1.58 (1.38, 1.81)	2.54, 2.11

**Table 10:** Ordered Minimum e-Value List

No.	Minimum E-Value
1	1.07E+ 05
2	374.76
3	9.20
4	4.33
5	3.31
6	2.96
7	2.83
8	2.76
9	2.39
10	2.28
11	2.20
12	2.13
13	2.11
14	2.07
15	1.85
16	1.82
17	1.77
18	1.68
19	1.66
20	1.62
21	1.56
22	1.50
23	1.37
24	1.31
25	1.30
26	1.21
27	1.21
28	1.19
29	1.17
30	1.13
31	1.12
32	1.06
33	1.05

### Cannabis legal status

**Fig. 19:**
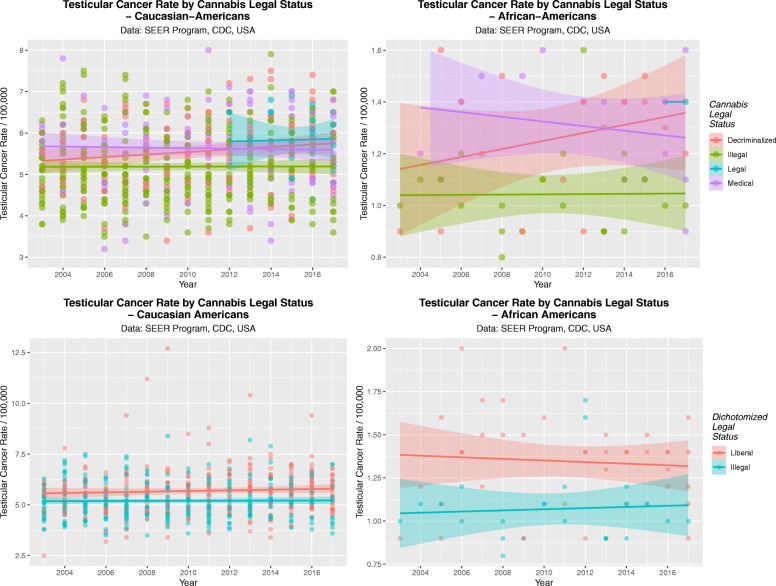
Scatterplots by time for the effects of cannabis legal status on testicular cancer rates. (**A** and **C**) Caucasian-Americans, (**B** and **D**) African-Americans. (**A** and **B**) Legal Status; (**C** and **D**) Dichotomized legal status, dichotomized as illegal v. not illegal status

It was of interest to determine if cannabis legalization had an effect on the TCR by ethnic background. This data is presented in Fig. 19 as scatterplots for Caucasian-Americans (A, C) and African-Americans (B, D). Panels A and B present the TCR by legal status and in panels B and D legal status is dichotomized into liberal regimes v. illegal paradigms.

These data are assessed quantitatively in Table 11A and B where the effect of cannabis legalization is noted to be highly significant and deleterious in both ethnic backgrounds by linear regression (β-estimate = 0.09 (0.06, 0.12), *P* = 6.5 × 10^− 10^ and (β-estimate = 0.22 (0.12, 0.32), *P* = 4.4 × 10^− 5^ in Caucasian-Americans and African-Americans respectively) and by mixed effects regression (Medical cannabis: β-estimate = 0.62 (0.43, 0.80), *P* = 2.5 × 10^− 11^ and Liberal cannabis (not illegal) paradigm: β-estimate = 0.48 (0.34, 0.62), *P* = 5.8 × 10^− 11^).

**Table 11:** Linear Regressions on Cannabis Legal Status

Linear Regression							
**Parameter Estimates**			**Model Parameters**				
**Parameter**	**Estimate (C.I.)**	***P*-Value**	**S.D.**	**R-Squared**	**F**	**dF**	**P**
***lm(Testicular_Cancer_Rate ~ Legal_Status)***							
***Caucasian-Americans***							
Liberal	0.09 (0.06, 0.12)	6.5E-10	0.1770	0.0563	39.35	1642	6.50E-10
***African-Americans***							
Liberal	0.22 (0.12, 0.32)	4.40E-05	0.1961	0.2286	19.37	1,61	4.40E-05
Mixed Effects Regression							
**Parameter Estimates**			**Model Parameters**				
**Parameter**	**Estimate (C.I.)**	***P*-Value**	**S.D.**	**AIC**	**BIC**	**logLik**	
***Status***							
***lme(Testicular_Cancer_Rate ~ Legal_Status, Random_Effects = Race)***							
Medical	0.62 (0.43, 0.8)	2.5E-11	0.9544	1967.652	1994.985	−977.8262	
Decriminalized	0.33 (0.15, 0.51)	0.0004					
Legal	0.61 (0.24, 0.98)	0.0014					
***Dichotomized_Status***							
***lme(Testicular_Cancer_Rate ~ Dichotomized_Status, Random_Effects = Race)***							
Liberal	0.48 (0.34, 0.62)	5.8E-11	0.9580	1966.987	1985.22	−979.4937	

The applicable e-Values for this data is presented at the foot of Table 9.

**Fig. 20:**
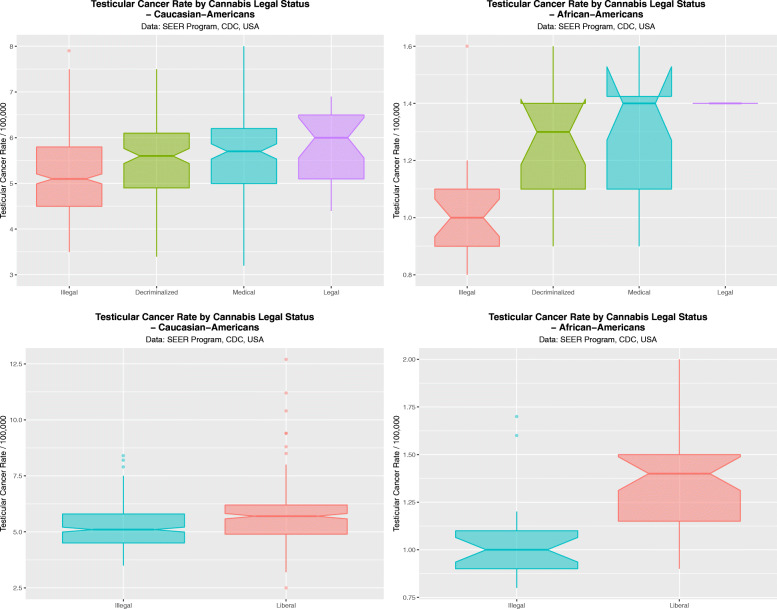
Boxplots by aggregated time for the effects of cannabis legal status on testicular cancer rates. (**A** and **C**) Caucasian-Americans, (**B** and **D**) African-Americans. (**A** and **B**) Legal Status; (**C** and **D**) Dichotomized legal status, dichotomized as illegal v. not illegal status

These data are aggregated by time and shown in Fig. 20. These data are significant for legal status in both races (Caucasian-Americans: ChiSqu.Trend = 179.2, df = 162, *P* = 0.0310; African-Americans: ChiSqu.Trend = 44.45, df = 30, *P* = 0.0434). When the data are dichotomized contrasting states where cannabis is illegal against those where it is not illegal these results become more significant (Caucasian-Americans: t = 6.1915, df = 538.95, *P* = 1.1820 × 10^− 9^; and African-Americans t = 4.50, df = 57.931, *P* = 3.327 × 10^− 5^).

## Discussion

### Main results

The present study assessed cannabis and other drug use as risk factors for testicular carcinogenesis and their potential to explain the well described ethnic differentials, and changes in TCR’s amongst ethnic populations across time. Data showed that exposure to THC and cannabigerol are risk factors for TC for all ethnicities investigated, and fulfil the criteria of causal relationships in all ethnicities studied. Data also confirmed the previously described four-fold elevation of TCR amongst Caucasian-Americans compared to African-Americans.

We confirm that time-based scatterplots and boxplots of intensity of cannabis use tend to follow TCR and the two are shown to be closely associated at multivariable regression by several different techniques. Different ethnicities demonstrate different sensitivities to the testicular oncogenic action of cannabinoids, and the pattern of TCR within each ethnicity is not necessarily constant e.g. Hawaii where it is much higher than elsewhere.

Since the relationships persist after inverse probability weighting and are accompanied by high e-Values, findings fulfil the quantitative criteria for causal relationships. These relationships were greatly strengthened when missing data are multiply imputed by chained equations. Legalization to make cannabis more available was also associated with higher TCRs. In that pro-cannabis legalization is associated with higher cannabis use and exposure [42] cannabis legalization may be said to exacerbate and contribute to higher TCRs.

### Biological and mechanistic considerations

#### Description of biology of NSGCT

TC is believed to arise due to genotoxic and epigenotoxic insults incurred during utero life on the germ stem cells which then become activated postnatally by the hormonal surge of puberty [1, 11–14]. Rising rates therefore may imply a rising incidence of some in utero genotoxic or epigenotoxic insult which becomes apparent only later in life.

The four cohort studies of the cannabis - TGCT relationship all described adult / adolescent cannabis exposure [6, 17–21]. This implies a very significant truncation of the usual time course of TGCT by excluding the period of in utero exposure. It is not explicit in our data whether the major aetiological exposure occurs in utero or in later life – or indeed if both may be implicated. In the case of NSGCT which is oncogenically de-differentiated backwards this implies significant and relatively rapid genomewide demethylation [14].

#### Ethnic differential

P53 is known as the “guardian of the genome” since it is widely connected across the genomic machinery to strongly shut down aberrant DNA replication in the presence of any form of genotoxic stress [43]. In this context it is worth describing the genomic elucidation of the above-mentioned ethnogenomic variability. Investigators intersected 62,567 genomewide association study (GWAS) cancer-associated single nucleotide variations (SNP’s) with 17,118 unique positive signals for P53 activation response elements (P53-RE’s) in four different cell lines using seven P53 activators [7]. The base sequences surrounding the 86 positive hits were compared to assess their fit with the two canonical decameric DNA recognition sequences in P53. At position rs4590952 in the kit P53-RE on chromosome 9 the position weight matrix value dropped from 15.6 to 11.1 (median = 13.8) with guanine to adenosine substitution. Three nearby sites have been identified in three previous GWAS screens as being associated with TCR and to have a three-fold elevation per allele in TCR risk amongst Caucasians [44–46]. The Kit - Kit-ligand dimer is the key nearby receptor ligand pair which acts as the master transcription factor for primordial germ cell, controls their specification and prevents differentiation, and is highly and uniquely expressed in seminomas rather than other TGCT’s. Kit also plays a key controlling role in haemopoietic stem cells and melanoblasts [7].

This mutation has been identified as a risk factor for both seminomas and non-seminomatous germ cell tumours [7]. This site is unique as it is activated by P53 activation rather than suppressed as is more usual [43]. In a subsequent assay in testicular tumour cell lines the per allele activation of the P53-RE by P53 activation was 188-fold (range 93 to 373- fold) [7]. This allele was positively selected for in seminomas (21.7-fold) and also amongst Caucasian-Americans. It was thought that the allele was positively selected for in light skinned races as its effect to stimulate melanoblast activation and proliferation in the tanning response to UV radiation was protective of the skin from UV induced carcinogenesis [7]. This was thought to explain is relative prevalence amongst light skinned races.

Other similar loci have also been described including rs995030 and research in this area is on-going at the present time [8].

#### Cannabinoid pathophysiology

High dose marijuana smoking has been shown to grossly disturb human sperm morphology with shrunken and bent sperm heads, bent tails, multiple tails, bilobed heads, tangled tails, multiple heads, pyknotic heads and polymorphonuclear pus cells all described [47].

In mice exposure to Δ9THC was shown to induce ring and chain chromosome formation with chains up to four chromosomes long due to end-to-end fusion formation [48].

These authors also showed that when mouse sperm were exposed to the cannabinoids Δ9THC, cannabinol and cannabidiol there was a dramatic increase in chromosomal translocations from about 1% at control levels, to 4.95–6.48% comparable to the positive control which was the cytotoxic drug mitomycin C at 6.73% [48].

Cannabinoids have been shown to have marked effects on sperm function including reduction of sperm concentration in seminal fluid, induction of DNA fragmentation, defective sperm maturation, disorders of DNA packing within sperm and protamine-histone replacement, DNA nicking in sperm by Tnp2, defective DNA repair, defects of nuclear size and incomplete DNA packing by failure of the histone-protamine transition [49–51].

Cannabinoids induce collapse of the inner mitochondrial membrane potential by several routes [50, 51].

THC exposure has been shown to lead to marked demethylation of the genome of human sperm [52] a change which makes genes more susceptible to genomic damage and chromosomal rearrangements [13, 16].

Cannabis exposure of both lymphocytes and oocytes has been shown to induce 20% cell death with a single division and marked chromosomal bridging and nuclear bleb formation in surviving cells [53]. Cannabinoid exposure has also long been known to be associated with micronucleus formation and comet tail formation, which are two of the major genotoxicity assays implicating chromosomal mis-segregation and single- and double- stranded DNA breaks respectively [15].

Moreover low micromolar doses of cannabidiol and its propyl analogue cannabidivarin have been shown to cause micronucleus formation and prominent comet tails on formal testing, changes which are greatly exacerbated in an oxidizing environment [15].

Downs syndrome has been linked with cannabis exposure in Hawaii, Colorado, Canada and Australia [54–57] and early termination of pregnancy for anomaly-corrected rates of Downs syndrome, trisomies 18 and 13, Turners syndrome and Deletion 22q11.2 in a space time and odds ratio analysis in the USA [58].

Prenatal cannabis use has been linked with acute lymphoid leukaemia which is primarily a disease characterized genetically by chromosomal translocations (unpublished data).

Adult cannabis exposure is linked with TC [17–20] and present study, which is itself caused by whole genome duplication, isochromosome 12 formation, deletion and augmentation of many chromosomal arms, over 1200 micro-RNA’s [13, 14] and genomewide DNA demethylation.

Formation of ring and chain concatenation of chromosomes in rodents was mentioned above [47].

This list implicates cannabinoids in major chromosomal toxicity by many mechanisms including chromosomal deletion, reduplication, megabase scale reduplications, longitudinal and transverse duplications and gene amplification and oncogenic cellular de-differentiation.

All of this demonstrates that cannabinoid-exposed cells are clearly genomically stressed.

In the context of genomic stress P53 is activated – and the ethnogenomic differential mechanism described above becomes activated as a stimulus to tumour cell proliferation in light skinned races, and to an onco-protective block to mitosis and meiosis in darker skinned races.

Given the aforementioned, it is possible that cannabis exposure causes in utero germ cell damage which is associated with TC, suggesting that cannabis use during pregnancy should be cautioned. This is consistent with recommendations by both the American College of Obstetrics and Gynaecology (ACOG) and the American Academy of Pediatric (AAP) [59–63]. Notwithstanding this advice, a significant number of American women are reported as using cannabis whilst pregnant, perhaps explaining part of the rise in TC across many communities. In Colorado 69% of cannabis dispensaries contacted by a group of researchers recommended cannabis use to pregnant women [64], while in California 24% of pregnant teenagers either self-admitted to cannabis use whilst pregnant or tested positive for it during their gestation [65]. Nationwide it was estimated in 2017 that 161,000 pregnant women used cannabis whilst pregnant [24].

It is however important to stress that while TC is generally believed to arise as a result of in utero germ cell anomalies which may be impacted on by maternal cannabis use, all published studies on the association have identified an association between TC and personal cannabis use, suggesting that one or more likely gene / epigenome - environment interactions are at play whereby postnatal and adult cannabinoid exposure contribute to underlying genetic risk as environmental causal and exacerbating factors. Since the epigenetic state of the primordial germ cells / gonocytes is a key determinant of the differentiation block experienced by all NSGCT tumour cells, it follows that part of the effect of postnatal cannabinoid exposure must be to de-differentiate susceptible cells into a more immature foetal-like and pro-oncogenic state.

### Generalizability

We feel that study findings are generalizable for many reasons. The SEER Cancer data is registry controlled and comes from most USA states and thus represents the great preponderance of the data from the population. The NSDUH survey has a good response rate at 74.1%. Moreover the effects we describe are often very strong. There is great internal consistency across results within this study with similar results being found for all ethnicities studied, and also good external consistency with all the published literature in this field. Moreover since data fulfil the criteria for causality we would expect that this causal relationship to hold widely across space and time.

### Strengths and limitations

This study has a number of strengths and limitations. Its strengths include the use of a large population dataset and registry controlled data, and a variety of advanced statistical methods including inverse probability weighting, mixed effects models, robust regression, two-step instrumental variable regression, multiple imputation of missing data by chained equations and e-Values. Further, many levels of significance are very high as are their corresponding e-Values. Since the relationships described amply fulfill the quantitative criteria for causality we feel that the relationships described herein are transportable to other situations and other times. The main limitation of the present work is the absence from this dataset of individual exposure data which is a limitation commonly shared with most epidemiological studies. Also spatiotemporal data on known risk factors such as cryptorchidism, inguinal herniae, industrial pollution and sedentary lifestyles was not available to the present investigators. It would be a useful advance if future studies could be repeated with these factors included in the multivariable analysis.

## Conclusion

Data analysis indicate that exposure to THC and cannabigerol is a risk factor for TC for all ethnicities. We have confirmed the four-fold elevation of TCR amongst Caucasian-Americans compared to African-Americans and data indicate that a likely gene-environment interaction is at play with cannabis the most likely environmental causal factor. In view of the high e-Values demonstrated we feel that the place of cannabis and cannabinoids is unlikely to be supplanted by other covariates with further research. All ethnicities are subject to an increase in testicular oncogenesis under a paradigm of increasing cannabinoid exposure with some ethnicities demonstrating marked differences in their apparent sensitivities. Time based plots and box pots of cannabis use and TCR generally move in parallel. Effects of cannabis, ethnic THC exposure and cannabinoid exposure are statistically highly significant, confirmed with a variety of multivariable techniques, and are independently significant. These relationships are strengthened by multiple imputation of missing ethnicity data. Findings fulfil the criteria of causal relationships in all ethnicities studied. Cannabis legalization significantly elevates the TCR for both African-American and Caucasian-American patients. In short, we feel that these findings are robust, fulfil the criteria for causal relatoinships and add an important transgenerational dimension to the present cannabis debate which applies to the major ethnic groups identified within the USA for which data is available.

## Data Availability

No permissions are required to access the data which was used and collated in this study, e.g. NSDUH study. Data including shapefiles and R programming script is made publicly available on the Mendeley Data Archive at this URL: 10.17632/yxy3dg2wt6.1 .
